# *TNFRSF10C* methylation is a new epigenetic biomarker for colorectal cancer

**DOI:** 10.7717/peerj.5336

**Published:** 2018-09-13

**Authors:** Cong Zhou, Ranran Pan, Haochang Hu, Bin Li, Jie Dai, Xiuru Ying, Hang Yu, Jie Zhong, Yiyi Mao, Yihan Zhang, Dongping Wu, Shiwei Duan

**Affiliations:** 1 Medical Genetics Center, School of Medicine, Ningbo University, Ningbo, China; 2 Department of Medical Oncology, Shaoxing People’s Hospital, Shaoxing Hospital of Zhejiang University, Zhejiang, China

**Keywords:** Colorectal cancer, Promoter, DNA methylation, *TNFRSF10C*

## Abstract

**Background:**

Abnormal methylation of *TNFRSF10C* was found to be associated with different types of cancers, excluding colorectal cancer (CRC). In this paper, the performance of *TNFRSF10C* methylation in CRC was studied in two stages.

**Method:**

The discovery stage was involved with 38 pairs of CRC tumor and paired adjacent non-tumor tissues, and 69 pairs of CRC tumor and paired adjacent non-tumor tissues were used for the validation stage. Quantitative methylation specific PCR (qMSP) method and percentage of methylated reference (PMR) were used to test and represent the methylation level of *TNFRSF10C*, respectively. A dual-luciferase reporter gene experiment was conducted to evaluate the promoter activity of *TNFRSF10C* fragment.

**Results:**

A significant association of *TNFRSF10C* promoter hypermethylation with CRC was found and validated (discovery stage: 24.67 ± 7.52 vs. 3.36 ± 0.89; *P* = 0.003; validation stage: 31.21 ± 12.48 vs. 4.52 ± 1.47; *P* = 0.0005). Subsequent analyses of TCGA data among 46 pairs of CRC samples further confirmed our findings (cg23965061: *P* = 4E − 6; cg14015044: *P* = 1E − 7). Dual-luciferase reporter gene assay revealed that *TNFRSF10C* fragment was able to significantly promote gene expression (Fold change = 2.375, *P* = 0.013). Our data confirmed that *TNFRSF10C* promoter hypermethylation can predict shorter overall survival of CRC patients (*P* = 0.032). Additionally, bioinformatics analyses indicated that *TNFRSF10C* hypermethylation was significantly associated with lower *TNFRSF10C* expression.

**Conclusion:**

Our work suggested that *TNFRSF10C* hypermethylation was significantly associated with the risk of CRC.

## Introduction

Colorectal cancer (CRC) is the third most common cancer and the fourth most universal cause of cancer-related death around the world (Ferlay et al., 2015). CRC has an annual incidence of 1.2 million new cases and 600,000 deaths (Chen et al., 2014). Though advanced screening and diagnostic technologies have developed continuously (Buturovic, 2014; Kim et al., 2008; Wang et al., 2014), the outcomes for CRC patients remain poor, and their average survival time is less than 30 months (Scartozzi et al., 2014).

Genetic and epigenetic studies are hot topics for CRC research (Fukushige & Horii, 2013; Okugawa, Grady & Goel, 2015). CRC was found to be relevant to aberrant expression of microRNAs (Mohammadi, Mansoori & Baradaran, 2016), altered histone modifications (Gargalionis et al., 2012), and disrupted regulation of inflammation (Janakiram & Rao, 2014). DNA methylation is one of the important epigenetic modification mechanisms (Dawson & Kouzarides, 2012), and it has been verified to be associated with CRC (Capuano et al., 2015).

Tumor necrosis factor receptor superfamily member 10c (*TNFRSF10C*) is located on 8p21.3 (23.01∼23.03 Mb), which is one of the most frequently deleted loci in CRC (Chughtai et al., 1999). *TNFRSF10C* functions as one of the TNF-related apoptosis inducing ligand-like (*TRAIL*) decoy receptors that could inhibit the intracellular signaling pathway of apoptosis (Cheng et al., 2009; Van Noesel et al., 2002). Additionally, *TNFRSF10C* expression was often found to be down-regulated in CRC (Macartney-Coxson et al., 2008), and a decreased *TNFRSF10C* copy number was shown to accelerate CRC distant metastasis (Tanenbaum et al., 2016). *TNFRSF10C* hypermethylation was found in mutiple cancers, including glioblastomas (Vaitkiene et al., 2013), breast cancer (Tserga et al., 2012), basal cell carcinomas (Stamatelli et al., 2014), melanoma (Venza et al., 2013), gastric cancer (Dauksa et al., 2014), hepatocellular carcinoma (Shin et al., 2010), pancreatic adenocarcinoma (Dauksa et al., 2012), ovarian neoplasia (Braga Lda et al., 2012), cervical cancer (Narayan et al., 2016), pheochromocytoma (Margetts et al., 2005), cholangiocarcinoma (Amornpisutt, Sriraksa & Limpaiboon, 2012), choroid plexus papilloma (Michalowski et al., 2006), and prostate carcinoma (Hornstein et al., 2008). Meanwhile, accumulating studies showed that *TNFRSF10C* hypermethylation might play an important role in tumorigenesis and tumor progression (Shivapurkar et al., 2004).

However, there was no literature about whether *TNFRSF10C* hypermethylation was associated with CRC. In light of previous findings, we carried out a two-stage study in order to investigate the performance of *TNFRSF10C* hypermethylation in CRC.

## Material and Methods

### Samples collection and ethics statement

**Figure 1:**
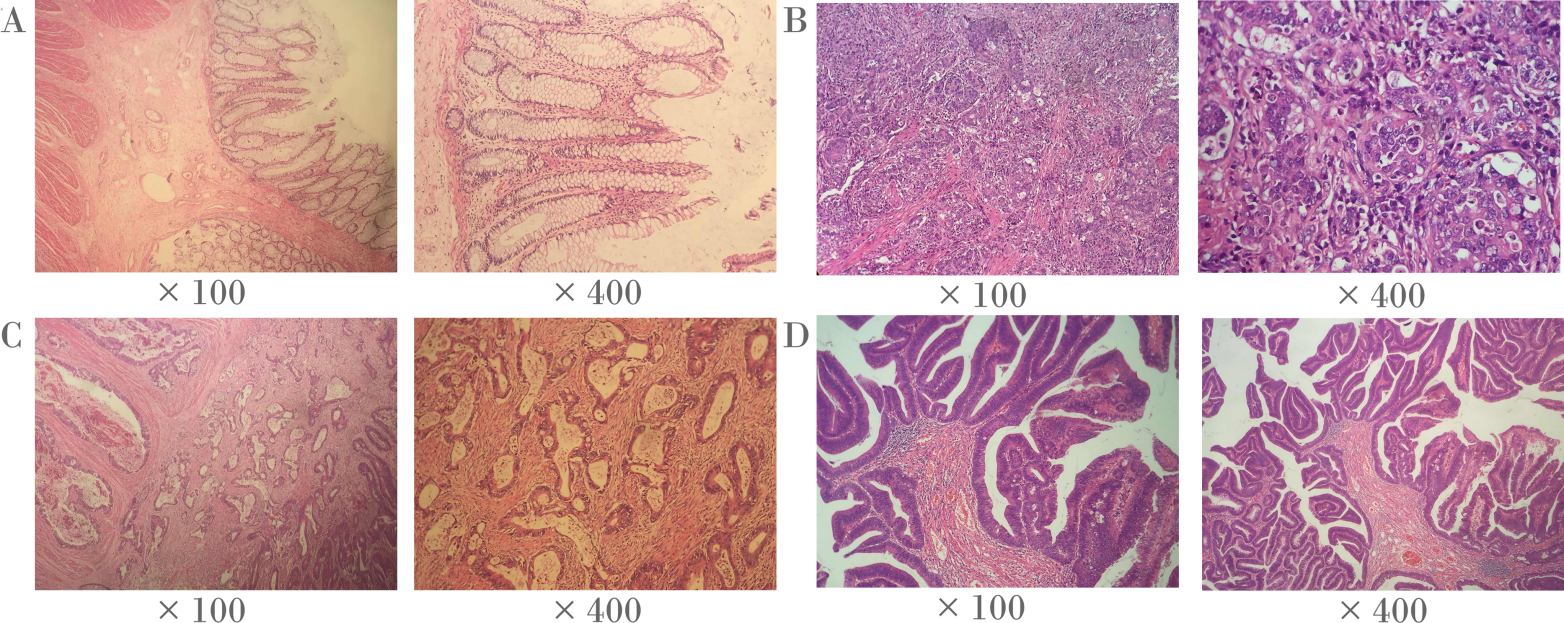
Representative histopathological photographs. (A) Normal colorectal tissues. (B) Poorly-differentiated CRC tissues. (C) Moderately-differentiated CRC tissues. (D) Well-differentiated CRC tissues.

Frozen tumor tissues and paired adjacent non-tumor tissues (5 cm away from the tumor) were collected from 38 CRC patients from the Third Affiliated Hospital of Nanjing University of Traditional Chinese Medicine (Jiangsu province, China) in the discovery stage. Frozen tumor tissues and paired adjacent non-tumor tissues (5 cm away from the tumor) of 69 CRC patients were collected from Zhejiang Tumor Hospital (Zhejiang province, China) and Shaoxing First People’s Hospital (Zhejiang province, China) for the verification. The corresponding clinical information was obtained at the time of surgery. All patients were diagnosed by pathological examination. No radiotherapy or chemotherapy was performed before surgery. We also used slides stained with hematoxylin and eosin (H & E) to identify representative areas of invasive tumors (Fig. 1). Over 80% of tumor cells were found in CRC specimens, and there were no tumor cells in the 5 cm adjacent non-tumor specimens. In fact, the majority of CRC tumors are adenocarcinoma (96%), and other histological types are rare, including signet ring cell carcinoma, squamous cell carcinoma, undifferentiated tumor and myeloid adenocarcinoma (Ponz de Leon & Di Gregorio, 2001). It is noteworthy that all CRC tumors in this study were adenocarcinomas. All the patients in the present study had signed informed consent, and this study was approved by the ethics committees of the above hospitals and Ningbo University.

### DNA extraction and bisulphite conversion

We strictly followed the instructions of the EZNATM Tissue DNA Kit (Omega Bio-Tek, Norcross, GA, USA) to extract the DNA from the tissue samples, and determined the DNA concentration using a Nanodrop 2000 spectrophotometer (Thermal Scientific Co. Ltd., Wilmington, MA, USA). The DNA solution was stored at −20 °C refrigerator for use. Genomic DNA was bisulphite converted using the EZ DNA Methylation-Gold Kit™ (Zymo Research, Orange, CA, USA). Generally, 500 ng of the original DNA was denatured by NaOH and the bisulphite was used to convert the unmethylated cytosine to uracil, while the methylated cytosine remains unchanged.

### Quantitative methylation specific PCR (qMSP)

Methylation-specific PCR (MSP) is a classic method to detect gene methylation, but it is error-prone as PCR product needs to be removed from tubes for further analysis, which tends to contaminate the work environment (Switzeny et al., 2016). Quantitative methylation specific PCR (qMSP) in the current study is a closed-tube technique which is one of the novel methylation quantitative methods.

In this study, a SYBR-GREEN based qMSP method was used to determine the gene methylation level of the sample. It has advantages such as rapidity, simplicity, low cost, and accurate quantification. *ACTB* was used as an internal reference. Normal human sperm DNA treated with SssI methyltransferase (Thermo Fisher Scientiific, Uppsala, Sweden) was used as a positive control, and enzyme-free water was used as a blank control. The qMSP assay uses bisulphite-modified DNA as a template to create a 20 µl qMSP system consisting of 10 µl of SYBR Green I Master mix (Roche, Basel, Switzerland), 0.5 µl of each of the upstream and downstream primers, and 1.0 µl of the DNA template, and the rest volumn was filed by ddH_2_O. The upstream qMSP primer of *TNFRSF10C* was 5′-AGGTGCGACCCAGCCCAG-3′, the downstream qMSP primer of *TNFRSF10C* was 5′-CGATAACGACGAACTT-3′, the upstream qMSP primer of *ACTB* was 5′-TGGTGATGGAGGAGGTTTAGTAAGT-3′, the downstream qMSP primer of *ACTB* was 5′-AACCAATAAAACCTACTCCTCCCTTAA-3′. The PMR (percentage of methylated reference) in each sample was calculated by 2^−ΔΔCt^ quantification approach (Kristensen et al., 2008). Specifically, the PMR of *TNFRSF10C* was calculated by this equation [PMR =2^−ΔΔCt^ ×100%, ΔΔCt = sample DNA (Ct_*TNFRSF*10*C*_–Ct_*ACTB*_) − fully methylated DNA (Ct_*TNFRSF*10*C*_–Ct_*ACTB*_)].

### Sanger sequencing and capillary electrophoresis of qMSP product

We randomly selected sodium bisulphite-modified DNA for Sanger sequencing. If compared with the original sequence, the uracil converted by bisulphite modification is completely converted to thymine, and the methylated cytosine remains unchanged, then the transformation process is verified thoroughly. In addition, the qMSP product was analyzed by a fully automated high resolution capillary electrophoresis apparatus (Bioptic, Taiwan, China) to verify that the fragment size of the product matches the theoretical fragment length.

### Dual-luciferase reporter gene assay

Previous studies indicated that a high correlation between methylation levels of neighboring CpG sites has been observed in CRC (Hu et al., 2017). Thus, the methylation profiles of adjacent CpG sites are often similar. To be noted, since the 85 bp fragment (nucleotides from +79 to +163 bp) in the methylation assay was too short to be constructed, we alternatively used a 485 bp fragment (nucleotides from −121 to +363 bp) that contains a 85 bp fragment (nucleotides from +79 to +163 bp) for the dual-luciferase assay. In accordance with UCSC Genome Browser, the selected fragment overlapped with multiple transcription factor binding sites, including CCCTC binding factor (CTCF) which played a vital role in gene regulation including promoter activation and repression (Rakha et al., 2004). For the above reasons, we chose the 485 bp fragment to test its promoter activity.

The human HEK293T cell line, obtained from the cell bank of the Chinese Academy of Sciences (Shanghai, China), was cultured and constructed recombinant plasmids. The fragment of *TNFRSF10C* promoter (−121 bp to +363 bp) was chemically synthesized. The cells were cultured on 24-well plates. After 12 h, 0.5 × 10^5^ cells per well were transfected with recombinant plasmid according to the manufacturer’s protocol (TransLipid HL Transfection Reagent, TransGen Biotech, Beijing, China). After 36 h of 293T cells transfection, we used SpectraMax 190 (Molecular Devices, Sunnyvale, CA, USA) to measure renilla and firefly luciferase activity. Reporter gene activity was assessed according to the manufacturer’s protocol (Dual-Luciferase^®^ Reporter Assay Systems, Promega, Madison city, WI, USA).

### Bioinformatics analyses

Methylation data from a total of 443 colorectal adenocarcinoma patients were extracted from the TCGA database (Methylation450k, https://genome-cancer.ucsc.edu/). Meanwhile, to evaluate the association between mRNA expression and *TNFRSF10C* methylation, an effective dataset of TCGA colorectal adenocarcinoma group containing 633 samples was downloaded from cBioPortal (http://www.cbioportal.org/). We compared on the expression changes of *TNFRSF10C* in two CRC cell lines (COLO320 and HT29) with and without 5′-AZA-deoxycytidine treatment, which derived from the Gene Expression Omnibus (GEO) database (http://www.ncbi.nlm.nih.gov/geo, accession no. GSE32323).

### Statistical analysis

Nonparametric Wilcoxon paired test was used to assess the methylation differences between tumor and paired adjacent non-tumor tissues. Nonparametric Wilcoxon paired test and paired samples *t* test were used to perform subgroup analysis of *TNFRSF10C* methylation and clinical characteristics. Spearman Correlation rank test was used to assess the correlation between *TNFRSF10C* methylation and *TNFRSF10C* expression. Furthermore, Kaplan–Meier survival analysis was implemented to assess the difference of overall survival between CRC patients with hypermethylated and hypomethylated *TNFRSF10C* promoters. A two-sided *P* < 0.05 indicated a significant difference.

## Results

In the current study, we recruited 107 CRC patients to study the relationship between *TNFRSF10C* methylation and CRC (Table 1). There were 30 prominent types, 72 ulcerative types, and five infiltrating types. The average age of CRC patients was 61 years (range: 28–86 years old).

**Table 1:** Clinical characteristics of CRC cases.

Variables	Stage-one experiment	Stage-two experiment	Total
Number	38	69	107
Age (years)	65.0 (57.0, 71.0)^a^	59.0 (52.0, 67.5)	61.0 (54.5, 71.0)^a^
Gender (F/M)	10/27^a^	24/45	34/72^a^
Tumor size (cm)	4.54 ± 1.49	4.94 ± 1.89	4.79 ± 1.77
Differentiation (Well/Poor)	34/4	55/14	99/18
Lymph node metastasis (+∕ − )	18/20	39/30	57/50
TNM stage (I + II/III + IV)	–	34/35	34/35
Pathological types (P/ U/ I)	8∕28∕2	22∕44∕3	30∕72∕5

**Notes.**

a Missing information for one case.

TITLE F stood for female and M stood for male Well comprised high and medium differentiation Poor comprised low and none differentiation P protrude type U ulcerative type I infiltrating type

**Figure 2:**
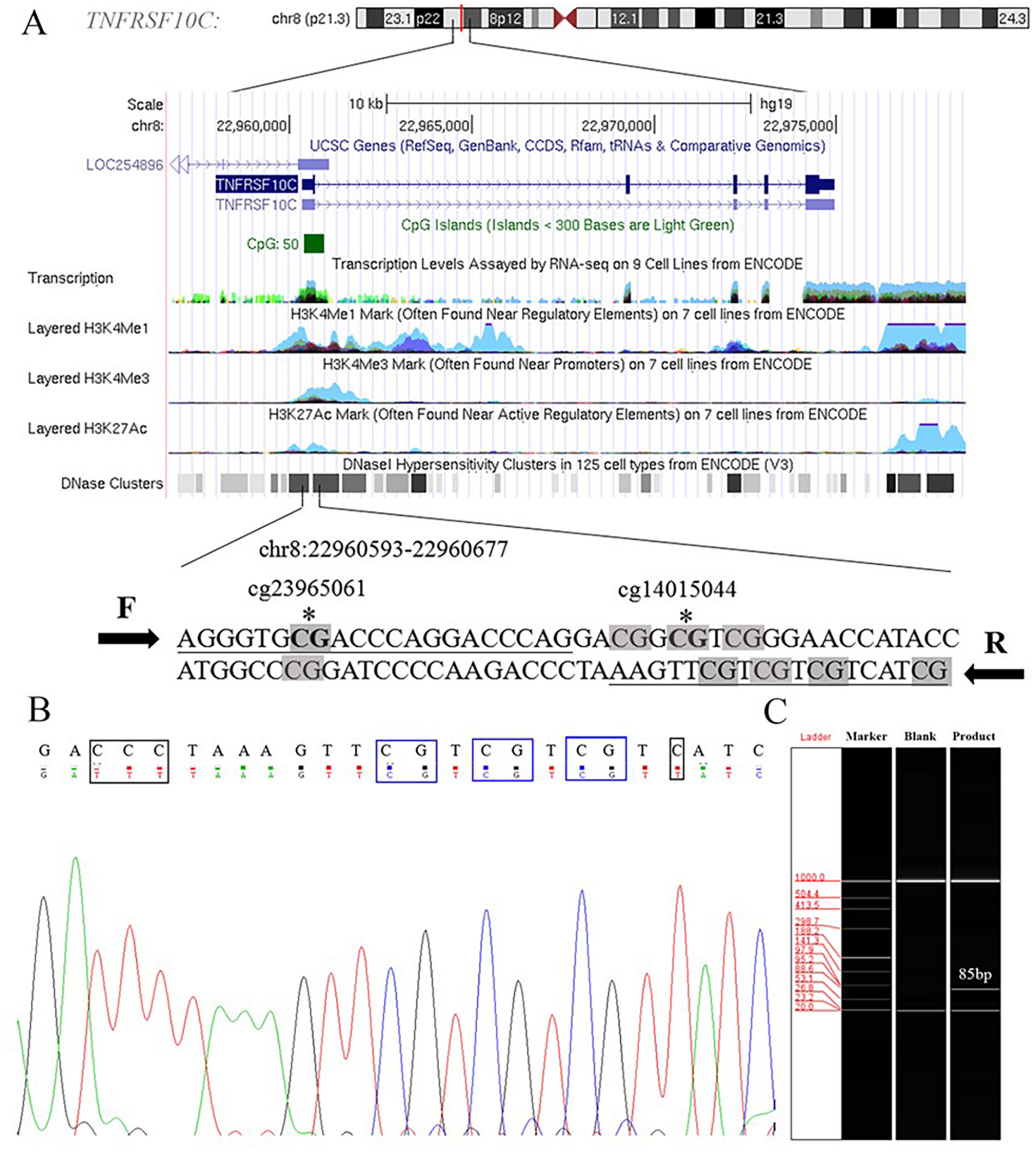
Primers and target amplification sequence in *TNFRSF10C* promoter CpG island (CGI) region. (A) F and R were forward and reverse primers, respectively. CpG sites in amplified sequences were signed in grey; Bold CpG sites marked with * were the probes (cg23965061 and cg14015044) detected in Methylation 450k microarray. (B) The top row of the sequence represents the original sequence of the target fragment, and the second row shows the converted sequences; CG dinucleotides which remained unchanged were outlined in blue, and C with corresponding converted T were outlined in black. (C) The result of capillary electrophoresis for amplification fragment (85 bp).

Aberrant methylation of promoter CpG island is associated with transcriptional inactivation of gene (Teodoridis, Strathdee & Brown, 2004). Since methylation often occurs at the position of CpG dinucleotides, we selected sites at the CpG island position in the promoter region (+79 ∼+163, 85 bp, chr8: 22960393–22960677) in the current study (Fig. S1). Two Methyl450 CpG sites (cg23965061 and cg14015044) in TCGA data were presented in the amplification fragment (Fig. 2A). Our Sanger sequencing result showed that the amplified fragment matched the target sequence and the bisulphite conversion was well performed (Fig. 2B). Capillary electrophoresis (Fig. 2C) verified that the length of amplified product was 85 bp as we expected.

**Figure 3:**
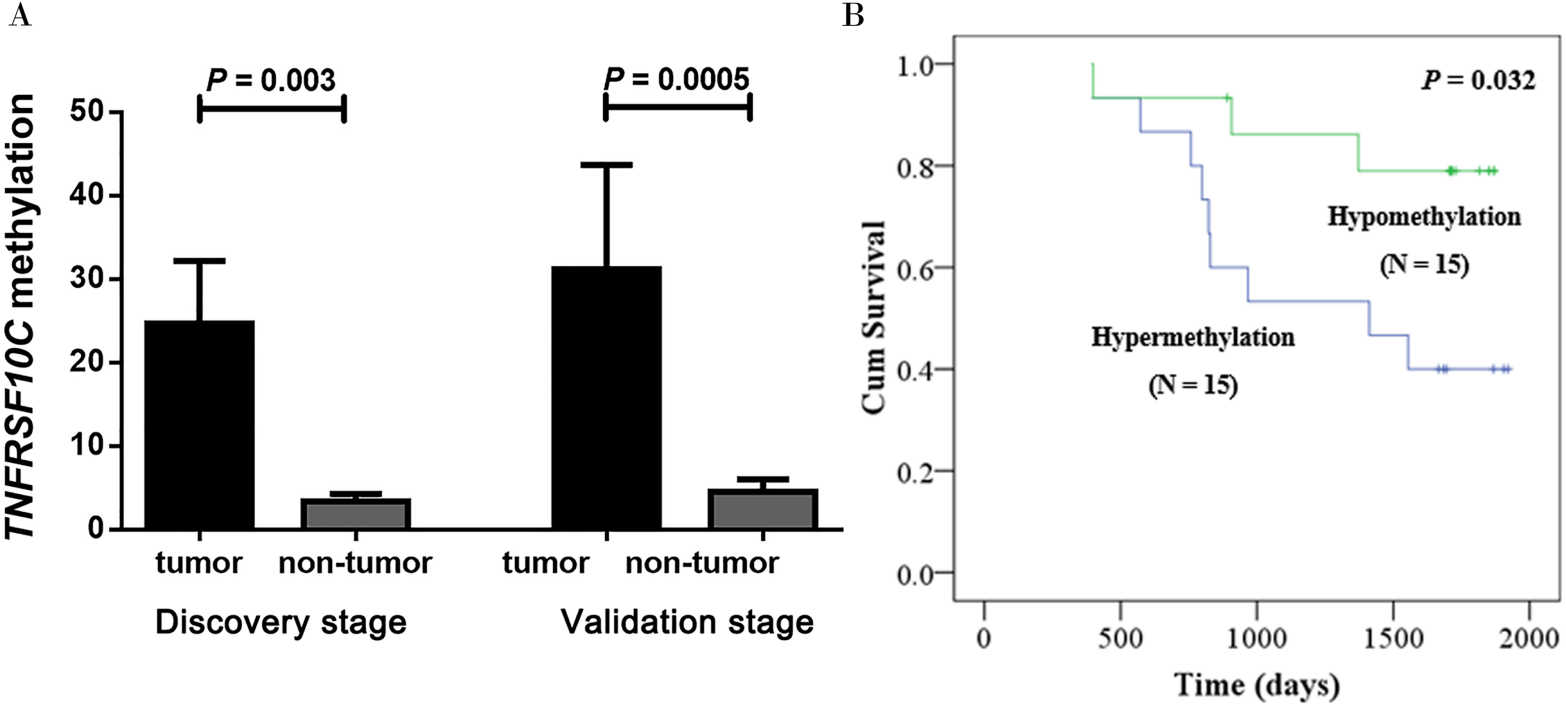
Hypermethylation of *TNFRSF10C* and its prognostic value. (A) Comparisons of *TNFRSF10C* methylation levels between tumor tissues and paired adjacent non-tumor tissues. The plot described as mean with SE. (B) The relationship between *TNFRSF10C* promoter methylation and overall survival in CRC patients.

Our two-stage association study comprised a total of 38 CRC patients in the discovery stage and 69 CRC patients in the validation stage. Our discovery-stage results showed that *TNFRSF10C* methylation was significantly increased in tumor tissues than in paired adjacent non-tumor tissues (mean PMR with standard error (SE), 24.67 ± 7.52 versus 3.36 ± 0.89, *P* = 0.003). And this association result was further confirmed in the validation-stage analysis (mean PMR with SE, 31.21 ±  12.48 in tumor tissues versus 4.52 ±  1.47 in paired adjacent non-tumor tissues, *P* = 0.0005, Fig. 3A).

Fortunately, there were 30 patients’ survival data obtained from the medical card in our cohort. Kaplan–Meier analysis was implemented to assess the prognostic effect of *TNFRSF10C* methylation in our cohort. Our results confirmed that CRC patients with methylated *TNFRSF10C* promoter had a poorer overall survival (OS) than those with unmethylated *TNFRSF10C* promoter (*P* = 0.032, Fig. 3B). Therefore, it could be a potentially clinical biomarker for better prognosis of CRC patients.

As shown in Table 2, we performed subgroup analysis between *TNFRSF10C* methylation and corresponding clinical information in CRC. Subgroup analysis was performed in the combined samples of discovery stage and validation stage. Our results revealed that the association of *TNFRSF10C* hypermethylation with CRC was specific to patients with TNM stage I + II tumors (*P* = 0.002) and patients with high and medium differentiation tumors (*P* = 4E − 5).

**Figure 4:**
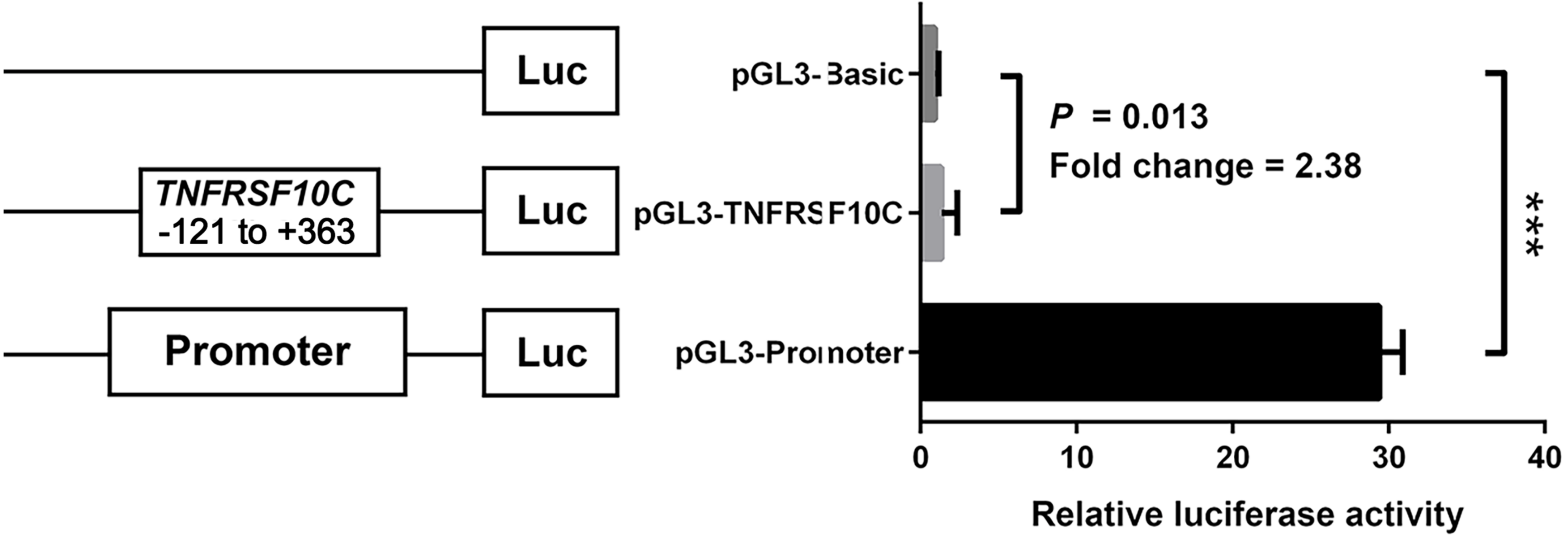
Dual-luciferase reporter gene assay in HEK-293T cell line. The pGL3-Basic and pGL3-Promoter vectors were constructed as negative and positive controls, respectively. Bars represented the means with standard deviations of three independent experiments.

We constructed a luciferase reporter vector containing synthetic *TNFRSF10C* promoter fragment. Our dual-luciferase reporter gene assay showed *TNFRSF10C* fragment (-121 bp to +363 bp) had a promoter activity, which was inferred by the comparison of reporter gene expression between insert-containing pGL3-basic vector group and pGL-3-basic vector group (Fold change = 2.375, *P* = 0.013, Fig. 4).

**Table 2:** Subgroup analysis of different clinical characteristics in total samples.

Variables	N (Pairs)	PMR (T)	PMR (N)	*P*
Differentiation				
High and medium	89	3.56 (0.35, 21.28)	1.64 (0.63, 3.31)	**4E−5**
Low and none	18	1.41 (0.39, 12.53)	1.23 (0.35, 2.85)	0.050
TNM stage				
I + II	34	5.27 (0.67.30.75)	1.49 (0.56, 3.35)	**0.002**
III + IV	35	1.09 (0.14, 11.05)	1.55 (0.40, 2.98)	0.095
Lymph node metastasis				
Positive	57	1.65 (0.26, 15.95)	1.63 (0.64, 3.41)	**0.009**
Negative	50	5.27 (0.57, 24.16)	1.58 (0.59, 2.89)	**0.0003**

**Notes.**

Stage-one experiment didn’t collect TNM stage information, with only 69 pairs of samples from stage-two experiment were enrolled for the subgroup analysis. PMR referred to the percentage of methylated reference. T referred to the group of tumors, N referred to the group of non-tumors. *P* value was calculated by nonparametric Wilcoxon paired tests. Bold value indicated a statistical significance.

**Figure 5:**
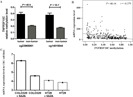
*TNFRSF10C* methylation and *TNFRSF10C* expression. (A) Methylation levels of cg23965061 and cg14015044 among 443 pairs of tumor and non-tumor tissues from TCGA colon and rectum adenocarcinoma database. The plot described as mean with SE. (B) Significant inverse correlation between *TNFRSF10C* methylation and expression among 633 individuals from TCGA colorectal adenocarcinoma database (*r* =  − 0.379, *P* = 4E − 14); (C) The changes of mRNA expression levels in a CRC cell line (SW48) with and without 5′-AZA-deoxycytidine treatment from GEO database (accession number GSE32323).

Additionally, TCGA data analysis confirmed a significantly increased *TNFRSF10C* methylation of the promoter fragment in CRC tumor tissues (cg23965061: *P* = 4E − 6, cg14015044: *P* = 1E − 7, Fig. 5A). All the above evidence indicated that *TNFRSF10C* hypermethylation could be a risk factor of CRC. Analyses among 633 TCGA colorectal adenocarcinoma samples showed a significantly inverse correlation between *TNFRSF10C* methylation and *TNFRSF10C* expression (*r* =  − 0.379, *P* = 4E-14, Fig. 5B). Further data mining of GEO data indicated that *TNFRSF10C* expression was significantly increased in two CRC cell lines after 5′-AZA-deoxycytidine treatment (COLO320, fold change = 1.36; HT29, fold change = 1.06, Fig. 5C).

## Discussion

In the present study, we explored the association between *TNFRSF10C* methylation and CRC in the Chinese population. By carrying out a two-stage study, we found a significant association between *TNFRSF10C* hypermethylation and CRC. Similarly, analysis of TCGA database supported that *TNFRSF10C* was significantly hypermethylated in CRC tumor tissues.

Although abnormal methylation of *TNFRSF10C* has been reported in other human cancers, our study has the following advantages. First, most of the previous studies used MSP, a technique that is difficult to quantify DNA methylation levels (Malpeli et al., 2011). In this study, we applied a qMSP method that is more suitable for molecular diagnostics (Hibi et al., 2011). Previous studies showed that gastric cancer (GC) and CRC shared several molecular characteristics, including abnormal methylation of tumor suppressor genes, microsatellite instability, and gene mutations (Hu et al., 2017). Dauksa et al. (2014) showed that the average methylation level of *TNFRSF10C* was significantly higher in GC patients than in the control group. However, there is no study on *TNFRSF10C* methylation in CRC. In this study, a two-stage study was performed to test the association of *TNFRSF10C* methylation with CRC, thus improving the reliability of the conclusions. Finally, a dual luciferase reporter gene assay was used to evaluate the potential regulatory mechanisms of *TNFRSF10C* methylation on gene expression. In conclusion, our study provides a better understanding of the relationship between *TNFRSF10C* methylation and CRC.

TNM stage is the most important prognostic factor for patients with CRC (Wei et al., 2015), and stage I+II often indicates the early stage with a favorable prognosis (Compton et al., 2000). Additionally, subgroup analyses by clinical phenotypes indicated that the association of *TNFRSF10C* hypermethylation with CRC was specific to patients with TNM stage I+II tumors. Therefore, *TNFRSF10C* hypermethylation could be served as an early diagnostic biomarker for CRC. Moreover, differentiation levels of cancer cells are often inversely correlated with the malignancy of tumors (Jogi et al., 2012). Significant association of *TNFRSF10C* hypermethylation with CRC was only found in patients with high and medium differentiation tumors. Above all, we speculated that *TNFRSF10C* hypermethylation might be an early event occurring in CRC carcinogenesis.

In the current study, there were significant differences in survival rate between *TNFRSF10C*-hypomethylated and *TNFRSF10C*-hypermethylated patients. *TNFRSF10C* promoter methylation is shown to be a significant predictive factor. Future work with a larger number and in various ethnic groups is warranted in order to confirm that *TNFRSF10C* promoter methylation as a commonly candidate biomarker on prognosis prediction.

*TNFRSF10C* methylation inversely associated with mRNA expression (Cai et al., 2011; Cheng et al., 2009; Shin et al., 2010; Vaitkiene et al., 2013) and protein expression (Sriraksa et al., 2011; Venza et al., 2013). Due to the limited amount of tissues, sufficient mRNA could not be obtained for additional gene expression assay. And the relationship between *TNFRSF10C* methylation and expression should be verified in subsequent experiments. Subsequently, TCGA analysis of 633 CRC samples confirmed that *TNFRSF10C* methylation was inversely correlated with mRNA expression. Our dual luciferase reporter assay revealed that the fragment of *TNFRSF10C* in the methylation assay was able to promote gene expression. Promoter hypermethylation of protein coding gene often induces expression silencing (Mohn et al., 2008; Moore, Le & Fan, 2013). However, there are two potential mechanisms of *TNFRSF10C* in the pathogenesis of cancer. As a TRAIL receptor, *TNFRSF10C* primarily activates the NF-*κ*b pathway of cancer cells (Murphy, Perry & Lawler, 2008). The NF-*κ*B pathway is pro-apoptotic, which is implicated in the pathogenesis of many human malignancies (Gilmore et al., 1996). Therefore, we inferred that CRC could use the promoter CGI methylation to silence *TNFRSF10C* expression to obtain the proliferation ability of cancer cells. And another mechanism suggests that *TNFRSF10C* can inhibit apoptosis induction (Murphy, Perry & Lawler, 2008) by competing the binding of TRAIL with *TNFRSF10A* and *TNFRSF10B*. TNFRSF10C’s down regulation will reduce its resistance to apoptosis and represent a protective response to tumor progression (Cheng et al., 2009), which is contrary to our observation. Therefore, future research needs to explore the exact mechanism of *TNFRSF10C* methylation in the pathogenesis of cancer.

Previous prostate cancer-related studies showed that the *TNFRSF10C* promoter methylation or deletion could regulate its gene expression in a dose-dependent manner (Cheng et al., 2009). However, compared with tumor patients without *TNFRSF10C* deletion, the frequency of promoter CGI hypermethylation was not significantly increased in *TNFRSF10C*-deficient tumor patients. Therefore, we speculated that *TNFRSF10C* promoter methylation and *TNFRSF10C* deletion may have different regulatory mechanisms for gene expression. Unfortunately, our study was based on a candidate gene approach, and we focused only on the role of *TNFRSF10C* promoter CGI methylation in CRC. More studies should be conducted in the future to investigate the interaction between CGI methylation and deletion in the *TNFRSF10C* promoter in CRC.

In conclusion, our results suggested a significant association of *TNFRSF10C* promoter hypermethylation with CRC. *TNFRSF10C* hypermethylation might contribute to the decreased expression of *TNFRSF10C* in CRC. However, the exact mechanism between the aberrant methylation and gene silencing of *TNFRSF10C* in CRC should be explored in the future.

##  Supplemental Information

10.7717/peerj.5336/supp-1 Table S1 Original data in the current study LN refers to lymphatic metastasis.

10.7717/peerj.5336/supp-2 Figure S1 Schematic representation of the *TNFRSF10C* promoter region The *TNFRSF10C* transcription start site was marked with a +1 and an arrow. Our qMSP amplicon (+79 ∼+163) and CpG island (−130 ∼+413) were located in the *TNFRSF10C* promoter region.

## Additional Information and Declarations

### Data Availability

The following information was supplied regarding data availability: The raw data are provided in Table S1.
