# Supplementary figures and images for: TNFRSF10C methylation is a new epigenetic biomarker for colorectal cancer
Source: PeerJ. 2018 Sep 13;6:e5336. doi: 10.7717/peerj.5336 (PMC6139245; doi:10.7717/peerj.5336)

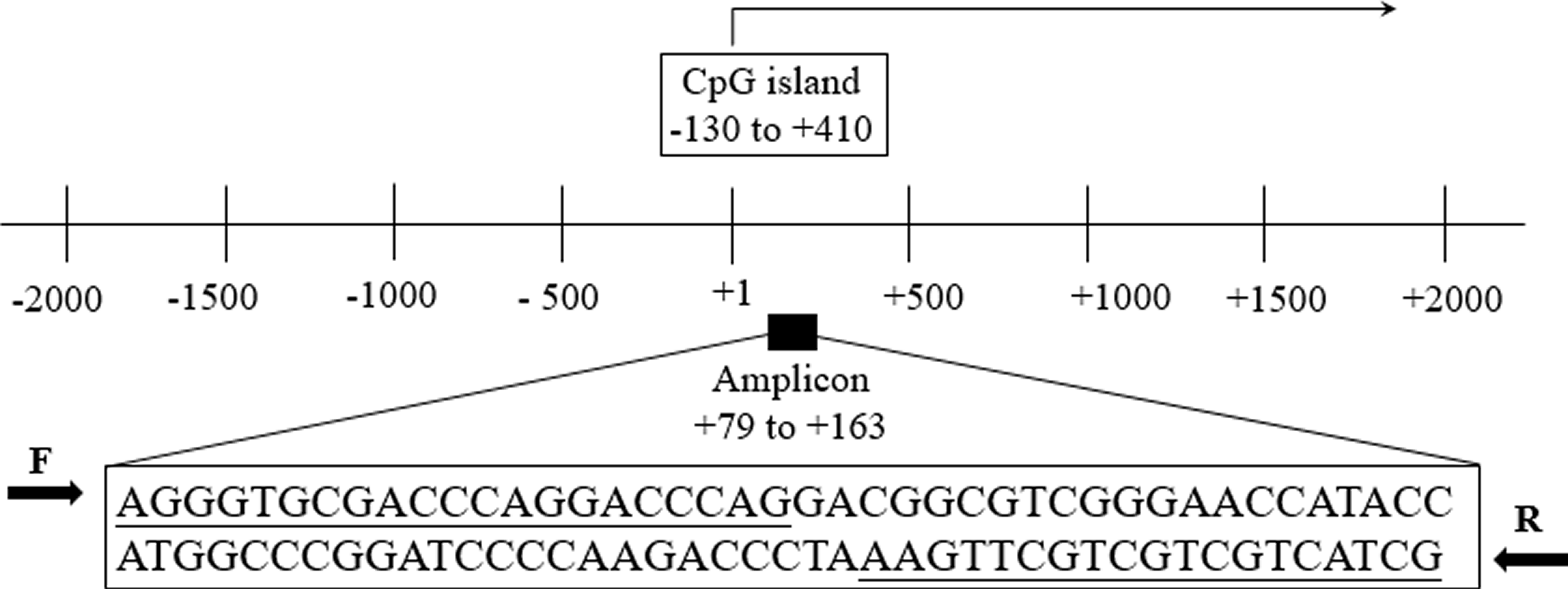

Supplement: Figure S1 — The TNFRSF10C transcription start site was marked with a +1 and an arrow. Our qMSP amplicon (+79 ∼+163) and CpG island (−130 ∼+413) were located in the TNFRSF10C promoter region. [file peerj-06-5336-s002.png]
